# PB.6. Management of radial scars on core biopsy

**DOI:** 10.1186/bcr3704

**Published:** 2014-11-03

**Authors:** L Hodkin, R Millican-Slater, N Sharma

**Affiliations:** 1 Leeds Teaching Hospitals Trust, Leeds, UK

## Introduction

Radial scar (RS) is a pathological diagnosis which is classified as B3 indeterminate due to the associated risk of malignancy. Further tissue sampling is therefore required, which traditionally was with surgical excision. In Leeds since 2009 we have been managing RS with vacuum-assisted biopsy, if <15 mm with no atypia; otherwise surgical management is taken. We reviewed cases since 2009 to identify how RS has been managed and if this is appropriate.

## Methods

From 1 April 2009 to 31 March 2013 all RSs assigned B3 on histology were identified. The imaging features, size, histology and subsequent management were documented.

## Results

Sixty-four patients were identified, 48 from breast screening and 16 symptomatic. Size of RS ranged from 2 to 70 mm. On imaging, five presented as asymmetry, one as asymmetry with calcification, 40 stromal deformities, five stromal deformities with calcification, four masses, eight calcifications and one normal. Overall, 13 RSs had associated atypia (three cancers) and 51 with no atypia on biopsy had six associated cancers on diagnostic surgery (RS >20 mm) - three LCIS and three DCIS. In total, 27/64 had diagnostic surgical biopsy (five had VAB preoperatively) and 37 had second-line VAB, of which seven are on 5-year FU.

## Conclusion

Yield of malignancy for small (<20 mm) RS with no atypia is low, therefore management with second-line VAB is appropriate rather than surgical diagnostic biopsy, providing there is radiological and pathological correlation with representative sampling.

RS with atypia still requires diagnostic excision, with a 23% upgrade to cancer discovered from our cases.

